# Effect of silver diamine fluoride on the microleakage of flowable resin composite and glass ionomer cement restorations to carious primary dentin: an-in vitro study

**DOI:** 10.1186/s12903-024-03861-2

**Published:** 2024-01-16

**Authors:** Sarah Osama, Amira Saad Badran, Basma Gamal Awad

**Affiliations:** Department of Pediatric Dentistry & Dental Public Health, Faculty of Dentistry, Ain Shams University, Cairo, Egypt

**Keywords:** Glass ionomer cement, Silver diamine fluoride, Flowable composite, Microleakage

## Abstract

**Background:**

This study aimed to evaluate the effect of Silver Diamine Fluoride (SDF) on the microleakage of flowable resin composite (FRC) and resin-modified glass ionomer cement (GIC) restorations bound to carious primary dentin.

**Methods:**

Forty-four extracted carious primary molars were allocated into four groups as follows (*n* = 11 teeth/group): Group I, Flowable resin composite (FRCa): SDF38% treatment + FRC, Group II, Flowable resin composite (FRCb): FRC without SDF treatment, Group III, Resin-modified glass ionomer cement (GICa): SDF38% treatment + GIC, Group IV, Resin-modified glass ionomer cement (GICb): GIC without SDF treatment. Specimens were subjected to thermo cycling at 500 cycles between 5 to 55 °C (dwell time of 60 seconds) in baths before being immersed for 24 h in a 1% toluidine blue solution. Microleakage testing was conducted for each specimen in two areas; occlusal and gingival. Specimens were evaluated under stereomicroscope at 4x magnification. Results were analyzed using Kruskal-Wallis test followed by pairwise comparisons utilizing Dunn’s post hoc test at *p* ≤ 0.05.

**Results:**

Insignificant differences between different groups (*p* = 0.49) were observed at the gingival walls area readings. The highest value was found in GICb (2.33 ± 0.52), while the lowest value was found in FRCa (1.71 ± 0.76). Insignificant differences between different groups (*p* = 0.982) were observed at the occlusal walls area readings. The highest value was found in FRCa (1.43 ± 0.98), while the lowest value was found in GICb (1.17 ± 1.33).

**Conclusion:**

SDF does not adversely affect the microleakage of FRC and GIC restorations bound to carious primary dentin.

## Background

Dental caries is a huge public health issue because it is one of the most frequent oral diseases in children. Despite the efforts undertaken in every country to prevent dental caries, it is still the most common illness in the world and the leading cause of tooth loss [1]. Dental caries is primarily caused by an ecological imbalance in the physiological equilibrium between tooth minerals and oral microbial biofilms [2].

Minimally invasive dentistry (MID) is a concept that combines prevention, remineralization, and minimal intervention for restoration replacement [3]. MIH applies the least invasive surgical approach to achieve treatment goals by removing as little healthy tissue as possible [4].

Silver Diamine Fluoride, or SDF, is an example of MID concept. It is unique in that it kills bacteria while also hardening the teeth, thereby arresting and preventing caries. It appears to be nearly twice as efficient at preventing caries as fluoride varnish [5]. SDF lowers dentin demineralization [6], promotes dental remineralization, increases biofilm pH, [7] and has an antibacterial impact against cariogenic bacteria [6, 8].

SDF is utilized in dentistry in numerous commercially available concentrations, including 12, 30, and 38%. The 38% SDF has been the most commercially used concentration nowadays as it has shown a significant effect in active caries arrest as well as prevention [9].

SDF plays a significant role in managing caries especially in children with high caries risk, medically compromised, those with behavioral challenges and those who have difficulty in accessing dental care [10]. SDF also has the ability to form a good biological seal at the restorative interface thus enhancing the prognosis of teeth treated with atraumatic restorative technique (ART) [11].

The mechanism of action of SDF relies in its ability to react with hydroxyapatite to generate fluorapatite, which prevents or lessens any future caries. When SDF is administered, it penetrates both the enamel and dentin, stocking the tooth with nearly twice as much subsurface fluoride as other fluoride forms [12]. Additionally, SDF specifically inhibits matrix metalloproteinases, cathepsins, and bacterial collagenases, which break down the exposed dentine organic matrix. Silver ions directly kill bacteria in lesions by disrupting membranes, denaturing proteins, and interfering with DNA replication. Almost any macromolecule can be deactivated by ionic silver. In terms of killing cariogenic bacteria in dentinal tubules, silver diamine fluoride outperforms other anti-caries medications [13].

However, SDF has a main adverse effect which is staining the caries lesion due to the silver compounds of the SDF. Because of this effect, the SDF has not been accepted widely. To decrease this negative effect, two alternatives exist, first one which is using the potassium iodide (KI) to mask staining and the other alternative is to apply glass ionomer or resin composite restoration over SDF to improve the aesthetics of the tooth after SDF application [12].

The application of the restorative material can be either done right away following the SDF in a single session or after two treatments of the SDF [14]. Yet, according to the Silver Modified Atraumatic Restorative technique “SMART”, it recommends the combination of two materials (SDF + Restorative material). This technique effectively stops the cavities and restores the tooth shape immediately which favors restoring both function and esthetics for primary teeth [15]. Nevertheless, this technique is usually conducted in children who are either medically compromised, uncooperative or in areas deprived of dental care.

The adhesiveness of any restorative material to the tooth structure is a significant factor in the selected restorative techniques. Any agent that is applied to dentine and enamel surfaces prior to restorative procedures could potentially interfere with the bonding [16]. which might in turn affect the sealing ability and might compromise the longevity of restoration.

Several authors investigated the effect of SDF on the microleakage of FRC and GIC restorations to permanent teeth. Yet, the studies that investigated the effect of SDF on the microleakage of FRC and GIC restorations to carious primary teeth are scarce. Therefore, the aim of the present study is to identify the effect of SDF as a caries arresting material on microleakage of FRC and GIC as filling materials in primary teeth. The null hypothesis was that there would be no effect when applying SDF prior to the restorations whether FRC or GIC in terms of microleakage [17].

## Methods

### Sample size estimation

A power analysis was designed to have adequate power to apply a statistical test of the null hypothesis that there is no difference would be found between different tested groups regarding microleakage score. By adopting an alpha (α) level of (0.05), a beta (β) of (0.2) (i.e., power = 80%), and an effect size (f) of (0.538) calculated based on the results of a previous study [18]; the required total sample size (n) was found to be (44) samples (i.e., 11 samples per group. )Sample size calculation was performed using G*Power version 3.1.9.7 [19].

### Specimens’ selection and preparation

Forty-four extracted primary molars with dentin caries and at least two sound surfaces of tooth structure remaining were selected. Teeth previously restored or severely destructed were excluded. Selected teeth were cleaned, polished with polishing paste and examined under a stereomicroscope (Olympus SZ1145, Olympus Optical Co. LTD, Tokyo, Japan) at a 40x magnification to exclude teeth with developmental defects, cracks. Teeth were stored in 0.9% sodium chloride solution at room temperature till use. A single experienced operator removed the infected carious dentin from the carious lesion by using hand excavator for standardization purposes. Surface was then evaluated with tactile confirmation that was defined as no “tug-back” sensation with a blunted explorer [20].

The teeth were randomly assigned into four groups according to each treatment protocol and restorative material received: (*n* = 11 teeth/group): Group I, Flowable resin composite (FRCa): SDF (FAgamin,Tedequim, Argentina) 38% treatment + FRC (Wave mv, SDI, North America, IL, USA). Group II, Flowable resin composite (FRCb): FRC without SDF treatment. Group III, Resin-modified glass ionomer cement (GICa): SDF 38% treatment + resin modified GIC (Fuji II LC, GC, CO Tokyo, Japan). Group IV, (GICb): Resin-modified GIC without SDF treatment.

### Treatment protocols

#### Group I: experimental group (Flowable resin composite) “FRCa”

A drop of 38% SDF was applied directly to each specimen using a micro-brush and allowed to act for 3 minutes, rinsed for 30 seconds using air/water spray then air-dried for 5 seconds [21, 22]. 35% phosphoric acid (Ultra-etch, Ultradent, UT, USA), was applied to each specimen for 15 seconds according to manufacturer’s instructions. Specimens were rinsed for 5 seconds by air/water syringe [23]. Specimens were air-dried. Bondfix (Voco, Cuxhaven, Germany) was applied to each specimen and allowed to act for 20 seconds then the adhesive layer was dried with an air jet for at least 5 seconds, light cured for 10 seconds using LED light curing system according to manufacturer’s instructions [24]. FRC (Wave mv, SDI, IL, North America) was applied and packed into the cavity using a carver and ball burnisher. Any excess material was removed using a carver. FRC was then light-cured for 40 seconds according to manufacturer’s instructions [25]. Polishing and Finishing was conducted to all specimens with low-speed handpiece and light pressure [26]. Similar steps were carried for Group II: Control group (Flowable resin composite) “FRCb” but without the application of 38% SDF.

#### Group III: experimental group (resin-modified glass ionomer cement) “GICa”

A drop of 38% SDF was applied directly to each specimen using a micro-brush and allowed to act for 3 minutes, rinsed for 30 seconds using air/water spray then air-dried for 5 seconds [21, 22]. Cavity conditioner was applied for 10 seconds to each specimen, washed off with water spray for 10 to 20 seconds. Specimens were then air-dried [26]. Resin-modified GIC (Fuji II LC, GC, Tokyo, Japan) was directly applied onto the specimen using carver and ball burnisher after the capsule was mixed for 10 seconds in the amalgamator at a speed of (+/− 4000 RPM). Any excess material was removed using a carver then light-cured using LED light curing system according to manufacturer’s instructions for 20 seconds [27]. Polishing and Finishing was conducted to all specimens with low-speed handpiece and light pressure [25]. Equia^Tm^ coat (GC, IL, USA) was then applied to the restoration and light cured for 10 seconds [27]. Similar steps were carried for Group IV: Control group (Resin-Modified Glass Ionomer Cement) “GICb” but without application of 38% SDF.

#### Thermo-cycling and dye application

**Fig. 1:**
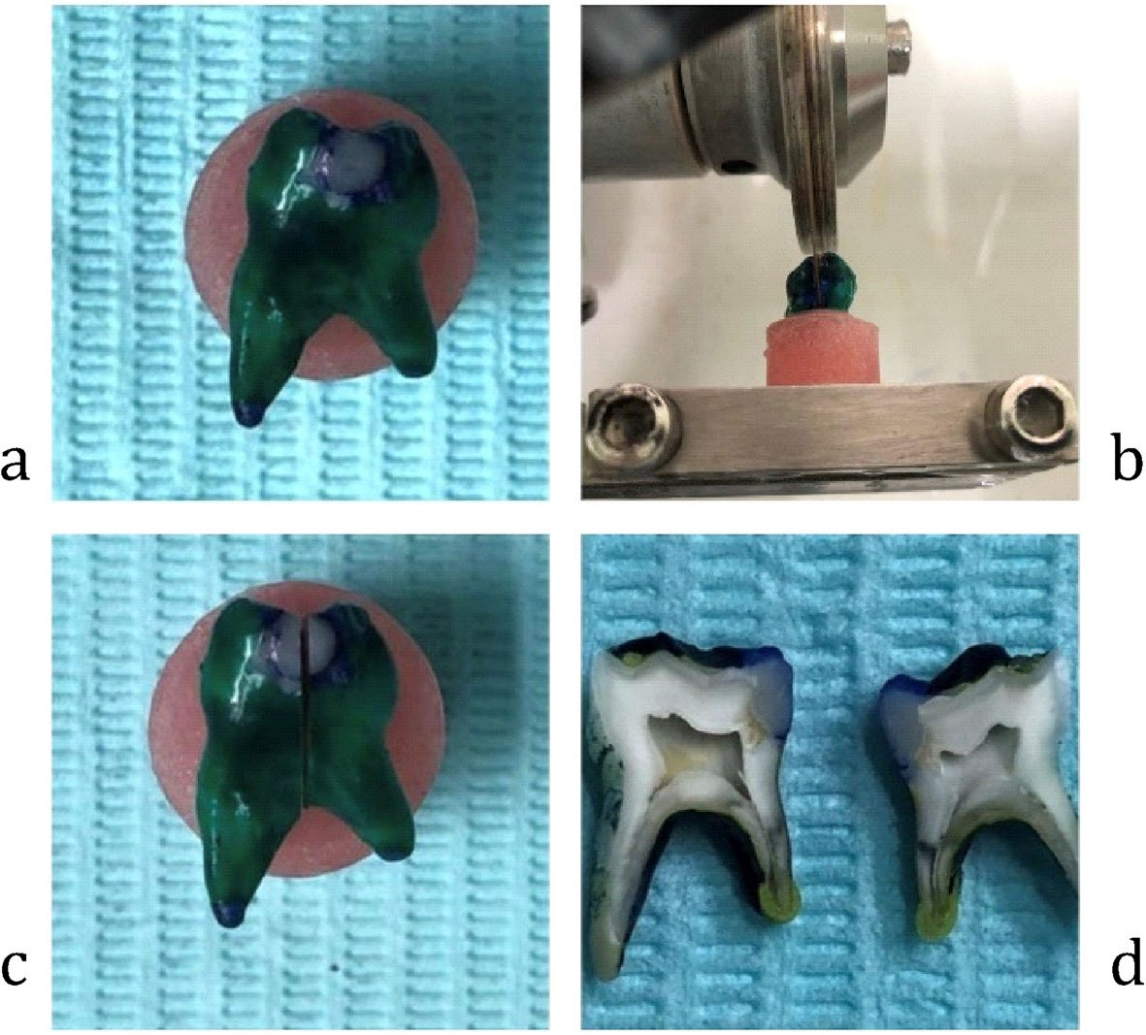
**a**-**d** Specimens were immersed for 24 hours in a 1% toluidine blue solution and sectioned buccolingually to be examined under stereomicroscope

All the specimens were stored in distilled water at 37 °C for 24 hours after finishing and polishing procedures [28]. The specimens were subjected to thermo cycling at 500 cycles between 5 to 55 °C (dwell time of 60 seconds) in baths [28]. Specimens were immersed for 24 h in a 1% toluidine blue solution [28] Fig. 1a.

#### Microleakage testing

**Fig. 2:**
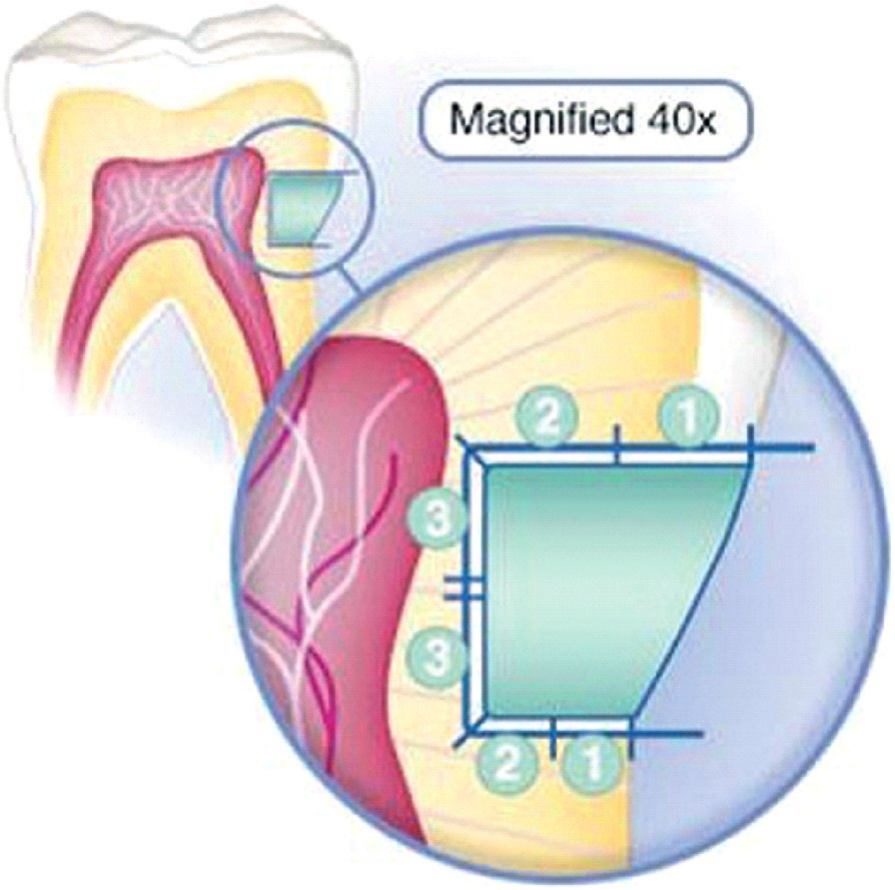
Diagram showing scores of dye leakage [30]

Specimens were fixed on prefabricated resin discs with a diameter of 12 mm and depth of 18 mm depth. Specimens were sectioned longitudinally in bucco-lingual direction using a diamond microsaw under water coolant (Isomet 4000, Buehler, IL,USA) [28] Fig. 1b, c, d. The degree of dye penetration in the occlusal and gingival walls was evaluated using a stereomicroscope (Olympus SZ1145, Olympus Optical Co. LTD, Tokyo, Japan) at 40x magnification. Scores were given to each tooth section whether occlusal or gingival, and the highest score was chosen to indicate the microleakage of each specimen. The high score from the two sections of each specimen was then recorded, representing the total amount of microleakage over the entire specimen to simplify the findings. Araujo CS, et al. [29] recommended criteria were used to record microleakage as shown in (Table 1) Fig. 2. A blind examiner carried out evaluations.

**Table 1:** Dye penetration Scores according to Araujo CS, et al. criteria [29]

Criteria	Scores
No leakage	0
Leakage extending to half the depth of cavity	1
Leakage extending to more than half of the depth of cavity	2
Leakage extending to the cavity floor	3

#### Statistical analysis

Ordinal data were represented as median and interquartile range (IQR) values and were analyzed using Kruskal-Wallis’s test. The significance level was set at *p* < 0.05 within all tests. Statistical analysis was performed with R statistical analysis software version 4.3.1 for Windows [31].

## Results

**Fig. 3:**
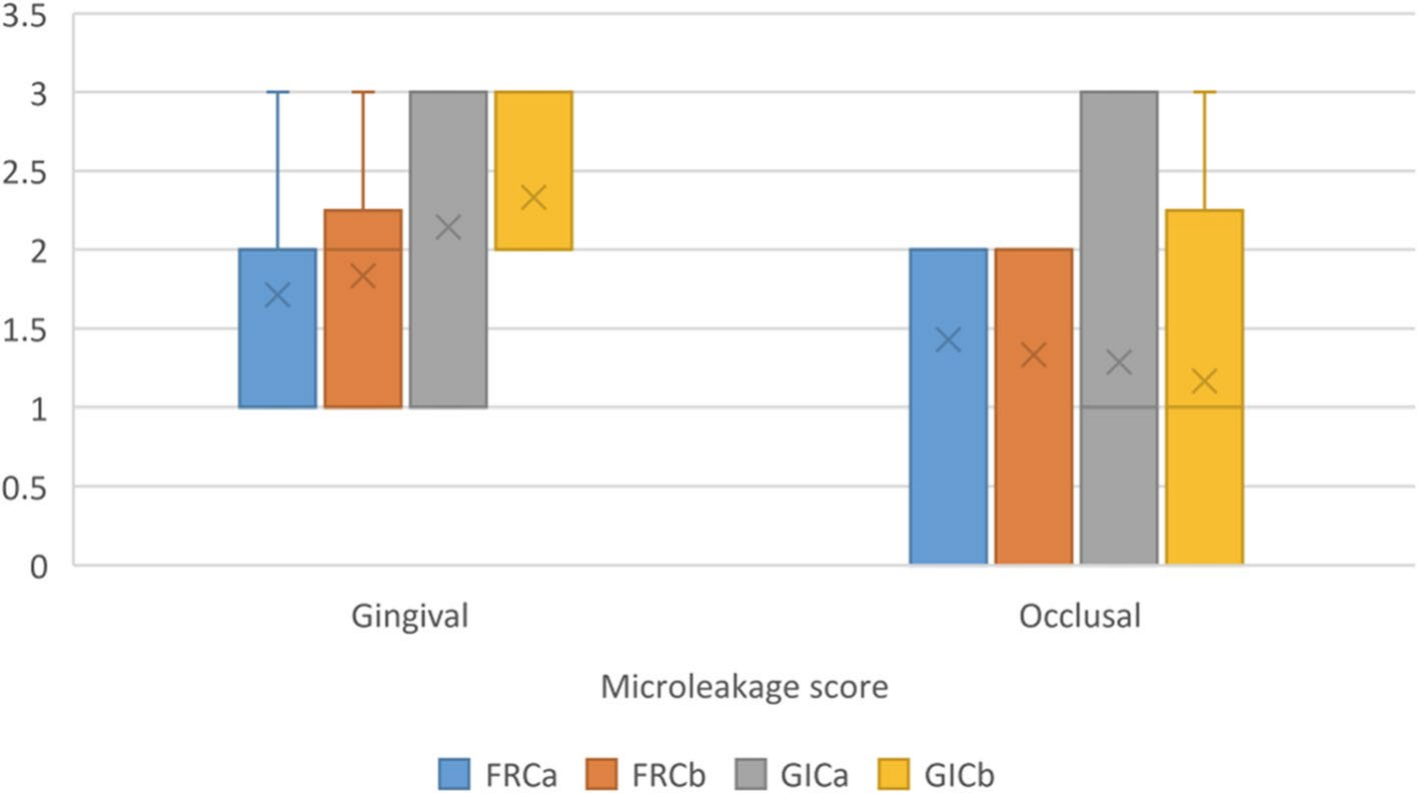
Box plot showing microleakage score values

**Fig. 4:**
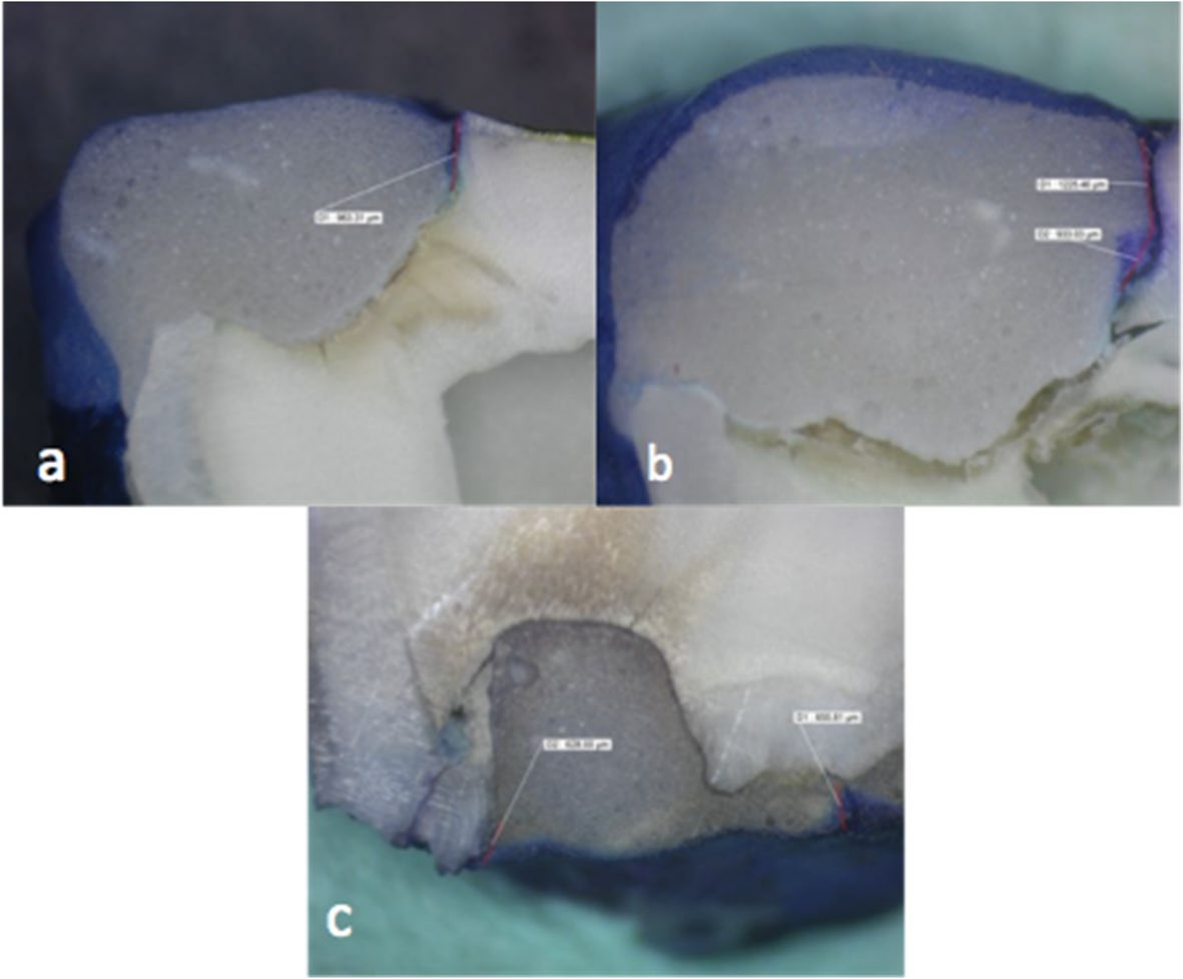
**a** Representing score 2 occlusal and score 0 gingival for dye penetration under stereomicroscope. **b** Representing score 3 occlusal and score 0 gingival for dye penetration under stereomicroscope. **c** Representing score 1 for both occlusal and gingival walls for dye penetration under stereomicroscope

Summary statistics and results of intergroup comparisons for microleakage score values are presented in Table 2 and in Fig. 3. Results showed for gingival measurements, the highest median score was found in GICa, while for occlusal measurements the highest values were found in FRCa and FRCb. However, for both measurements the difference between tested groups was not statistically significant (*p* > 0.05). Figure 4 demonstrates scoring images taken under the stereomicroscope.

**Table 2:** Summary statistics and intergroup comparisons of microleakage score

Position	Microleakage score [Median (IQR)]				χ^2^	*p*-value
	FRCa	FRCb	GICa	GICb		
*Gingival*	2.00 (1.00)	2.00 (0.75)	3.00 (2.00)	2.00 (0.75)	2.42	0.49
*Occlusal*	2.00 (1.00)	2.00 (1.50)	1.00 (1.50)	1.00 (2.00)	0.17	0.982

## Discussion

The prevalence & severity of caries are high among children in underdeveloped countries. The high expense of restorative care and the lack of understanding regarding oral health care are the complications that hinder the initiatives taken to control dental caries in such countries. Moreover, the sequelae of untreated dental caries has an impact on several spheres and facets of quality of life, including the overall well-being of the family, the social, functional, and emotional limitations of the child [32]. Many children undergo sedation or general anesthesia owing to their young age and lack of co-operation, which encounters families with a financial burden [33]. Fortunately, there are several ways of preventing dental decay such as fluoride varnish that can prevent cavities in young children in a safe and efficient manner. SDF is another form that works in a different method for prevention of tooth decay, particularly at the cavitation stage in preschoolers. SDF’s safety, effectiveness, feasibility, and ability to prevent and stop dentin caries may revolutionize pediatric and community dentistry and make a breakthrough as a dental agent for this century [34, 35].

SDF plays a significant role in managing caries especially in children with high caries risk, medically compromised, those with behavioral challenges and those who have difficulty in accessing dental care [10]. SDF also could form a good biological seal at the restorative interface thus enhancing the prognosis of teeth treated with atraumatic restorative technique (ART) [11].

Prior research indicated that SDF had both re-mineralizing and re-hardening effect on untreated carious lesions. Dentin treated with SDF has shown to have a higher mineral content than normal dentin [36].

It is proposed that the application of SDF on caries-affected dentin would raise the levels of fluoride, calcium and phosphate. Accordingly, when SDF interacts with hydroxyapatite, fluorapatite and insoluble silver phosphate is produced. In addition, the fluoride released from the SDF aids in deposition of silver phosphate which restores the mineral content of dentin resulting in hardening of soft affected dentin [37, 38].

As the use of silver diamine fluoride to stop cavitated lesions has grown in favor [39]. Its acceptance by some populations may be constrained by the exposed dentine’s black coloring brought on by the penetration of silver compounds into dentinal tubules [40]. To solve this problem, it has been suggested that we restore the tooth immediately after SDF application.

Several restorative materials serve as an adjunctive in the process ossf remineralizing demineralized teeth. The release of fluoride and other elements might be crucial in this remineralization process. Flowable resin composite especially Wave mv was used in the present study as it contains specially treated nano-fillers to maximize polishability, wear resistance and strength. Also, due to its fluoride release property where the fluoride ions diffuse from the resin into the surrounding tooth. This ionic movement is caused by oral fluid passing in and out of the resin and tooth, acting as a carrier for the fluoride ions [25]. Glass ionomer cement is another example of restorative materials that has the capability of forming strong bond to dental structures, biocompatibility, low shrinkage and the remineralization effect through constant fluoride release [41]. Such properties are essential for restoring primary teeth especially in children with high caries risk.

Proper seal of the cavity by having a good bond to the tooth structure is considered a main pillar for the longevity of any restoration and aids to prevent secondary caries [38]. The performance of the restorative material through its sealing ability could be tested through microleakage that is widely accepted and cheap [28].

There are different methods that can be used to evaluate microleakage such as the use of dyes, radioactive isotopes, air pressure, microorganisms, neutron activation analysis, artificial caries, scanning electron microscopy (SEM) [42–45]. Despite this, the use of dyes remains one of the most commonly used methods owing to its ease of use and low cost [46].

Fayyad and Shortall [47] used an image analysis tool connected to a stereomicroscope to evaluate dye penetration. It was possible to measure the precise depth of dye penetration along the interface using digital image microscopy. The length of the leakage at the dental restoration interface and the area of the leakage into the coronal dentin were measured by Glyn Jones J, et al. [48] utilizing dye penetration, 5.0% buffered eosin, and image analysis.

Results showed that, there was no statistical significant difference in the gingival and occlusal wall readings for microleakage test between 38%SDF pretreated carious primary dentin group and the control group without SDF application.

Results of this study came in accordance with Gupta J, et al. [49] who reported that silver diamine fluoride (SDF) pretreated premolar teeth did not have a significant difference in microleakage between resin modified GIC and tooth structure when compared to other groups not treated by SDF. Besides, Uzel I, et al. [28] found no significant difference in microleakage scores between the group of permanent teeth treated with SDF 38% before the application of resin composite and the group of teeth without SDF treatment.

Results came also in line with; Quock R, et al. [50] who found that SDF 38% treatment had no adverse effect on microleakage rates of GIC restorations in spoon-excavated primary molars neither at occlusal margin groups nor at gingival margin groups. Soliman N, et al. [17] also concluded that there was no significance difference between the dentin of primary teeth pretreated with SDF 38% group and the control group as regard to the microleakage and found no influence on the marginal seal of resin modified GIC to dentin of primary teeth.

In the present study, it was noticed that most dye leakage of specimens scored between score 1& 2 in the SDF pretreated group. This could be attributed to the fact that the dentinal tubules could be occluded by proteins precipitate formed by silver ions. Moreover, the reaction of fluoride ions with calcium ions forms a precipitate of calcium fluoride (CaF2) plugging the dentinal tubules [47].

On the other hand, the results of the present study were not consistent with Hassanen D, et al. [51]. This might be attributed to the different methodology, which involved a different cavity design as well as using a high-speed hand piece in caries removal [48].

Pérez-Hernández F, et al. conducted another study [18], where the impact of SDF treatment on the adhesion and microleakage of a pit and fissure sealant to tooth enamel was examined. Results showed significant difference when comparing the SDF treated samples with the non-treated SDF samples. Microleakage was (81.6%) in non-treated SDF group compared to microleakage SDF treated samples by (47%). It was concluded that treating a tooth surface with SDF prior to the application of fissure sealants decreases the microleakage of the pit and fissure sealant. These findings suggest that the application of pit and fissure sealant after SDF is a valid treatment decision and may represent an easier and effective option, with minor aesthetic alteration, as a minimally invasive treatment of caries and might be a further scope of research.

## Limitations of the study

The current study revealed some shortcomings. Due to its in-vitro nature, it cannot perfectly mimic the intra-oral environment, especially the varying pH. It was also challenging to identify the dark blue color of the methylene blue dye from the black stain brought on by SDF pretreatment of tooth structure in the microleakage test.

Therefore, care must be taken when applying direct interpretations to clinical situations due to the obvious limits of in vitro experiments.

Also, in this test, caries excavation was conducted using hand instrument (spoon excavator), which in clinical practice might not be sufficient and might be used in conjunction with other rotary instrument to smooth margins and remove unsupported enamel. Thus, the use of such rotary instruments might have an effect on microleakage test results. Also, standardization of carious removal is recommended in further research.

## Conclusion

It can be concluded that the pretreatment of carious primary dentin with SDF 38% does not adversely affect the marginal seal when restoring with FRC or resin modified GIC restorations.

## Data Availability

The datasets generated and/or analyzed during the current study are available from the first author “Sarah Osama” upon request.
